# Treatment adherence and associated factors among glaucoma patients attending Ophthalmic units of referral hospitals in North West Ethiopia, 2019

**DOI:** 10.3389/fopht.2022.985893

**Published:** 2022-11-03

**Authors:** Setarg Ayenew Birhanie, Girma Alem Getie, Mulugeta Tesfa, Henok Mulugeta, Mihretie Gedfew, Yeshimareg Shita Mekete, Tiliksew Liknaw, Mikiyas Muche Teshale, Melkamu Tilahun, Baye Tsegaye Amlak, Fentahun Minwuyelet Yitayew, Temesgen Ayenew, Bekalu Bewket, Dejen Tsegaye

**Affiliations:** ^1^ Debre Markos University, College of Health Science, Department of Nursing, Debre Markos, Ethiopia; ^2^ School of nursing and midwifery, faculty of health, university of technology, Sydney, NSW, Australia; ^3^ Debre Markos University, School of medicine, Department of Physiology, Debre Markos, Ethiopia; ^4^ Injibara University, College of Health Science, Department of Nursing, Injibara, Ethiopia

**Keywords:** glaucoma, treatment adherence, Ethiopia, Felegehiwot, Debremarkos

## Abstract

**Objective:**

Glaucoma is one of the common eye disorders resulting from optic neuropathy, which leads to irreversible blindness if left untreated. Poor adherence to glaucoma medical treatments typically leads to some serious consequences, such as progressive visual impairment and blindness. The aim of this study was to assess adherence to treatment and associated factors among patients with glaucoma attending at Northwest Ethiopia referral hospitals.

**Method:**

From March 1st to April 30th, 2019, an institution-based cross-sectional study was conducted on 382 consecutive glaucoma patients attending at Northwest Ethiopia referral hospitals. Data about adherence to glaucoma treatment was collected by using a standardized tool, the Morisky Medication Adherence Scale-8, through an interviewer-administered questionnaire. Each collected data set was coded and entered into Epi-Data version 4.2, and analysis was done by using STATA version 14.0 statistical software. A logistic regression model was fitted to assess the effect of an independent variable on the dependent variable. A p-value < 0.05 was considered to declare a statistically significant association. The study proposal was approved by the Debre Markos University ethical review committee.

**Results:**

Among the study participants, 189 (49.5%) were adherent to glaucoma treatment. In this study, occupation (farmer), good knowledge, favorable attitude, a short distance from patients’ homes to hospitals, and scheduling problems for glaucoma medical follow-up visits were significant factors associated with adherence to glaucoma treatments.

**Conclusion:**

The study has identified the adherence level as being low. Patient related factors and health care system related factors were significantly associated with adherence to glaucoma treatments. Appropriate patient education and planning a patient follow-up strategy might improve patients’ adherence to glaucoma treatment. Care providers should place emphasis on the importance of adherence.

## Introduction

Glaucoma is a group of ocular conditions characterized by optic nerve damage (1) that ultimately leads to irreversible blindness if left untreated (2).However, with early detection and treatment, it is possible to protect the eyes against serious vision loss (3, 4).

Worldwide, about 64 million people were affected by glaucoma in 2013 and this prevalence is expected to reach 76.0 and 111.8 million by 2020 and 2040, respectively (3).Glaucoma is the second leading cause of blindness globally (4). Glaucoma inexplicably affects more Africans and Asians (5) and it is considered as a public health problem in sub-Saharan Africa (6). In Africa, glaucoma accounts for 15% of blindness, and it is the region with the highest prevalence of blindness relative to other regions worldwide (7). Studies about glaucoma treatment adherence done in the US, Canada, and Dutch revealed adherence rates of 60%, 72.1% and 72.7% respectively (8–10).Another hospital-based cross-sectional studies conducted in Iran, North India and Egypt showed that 32.5%, 51.6%and 46.4% of glaucoma patients were adherent to their glaucoma treatment respectively (11–13). According to studies done in African countries, patients with glaucoma adhered to their medication at rates of 63.2% in Nigeria (14) and 60.1% in Ghana) (15). Other similar studies done in Ethiopia at Jima University Specialized Hospital and Menelik II Referral Hospital had identified the adherence rate of 32.5% and 42.6% (2, 16) respectively.

Adherence is generally defined as the extent to which patients take medications as prescribed by their health care providers (17).Self-report and healthcare professional assessments are the most common tools used to rate adherence to medication (18). The extent of the problem of non-adherence among patients with glaucoma is not adequately investigated in developing countries (16).

The most common risk factors for glaucoma include family history of glaucoma, African American race, older age, diabetes mellitus, cardiovascular disease, migraine syndromes, nearsightedness (myopia), eye trauma, and prolonged use of topical or systemic corticosteroids (19). Several studies suggest multiple reasons for poor adherence to glaucoma therapy. Those factors were themed as therapy-related factors, patient-related factors, health care system-related factors, and disease-related factors (16, 20, 21).

Review of literature realized that level of adherence to glaucoma medical treatment and associated factors have been poorly explored previously in Ethiopian researches, particularly in the selected study area. Therefore, the objective of this study was to assess adherence to glaucoma treatment and associated factors among patients with glaucoma attending at ophthalmic unit of referral hospitals in North West Ethiopia, 2019.

## Methods

### Study design and setting

An institution-based cross-sectional study design was conducted in Debre Markos comprehensive specialized hospital (DMCSH) and Felege Hiwot Comprehensive specialized hospital (FHCSH). The study was done from March 1st to April 30th, 2019, on 382 glaucoma patients who had follow up at the ophthalmic units of DMCSH and FHCSH. DMCSH and FHCSH are the referral hospitals in North West Ethiopia.

### Eligibility criteria

#### Inclusion criteria

All adult patients (> 18 years old) with glaucoma who had follow-up during the study period and who were on glaucoma medical treatment for at least two months before the commencement of the study were recruited.

#### Exclusion criteria

Those glaucoma cases with ophthalmic surgical procedures that did not require glaucoma medications were excluded from the study.

#### Sample size and sampling technique

The sample size was determined by using single population proportion formula with 95% confidence interval level, marginal error (d) of 5% and the prevalence (P) of 42.6% (16) and the final sample size including non-response rate was 414. Participants were allocated proportionally from the two hospitals based on the number of patients attending the ophthalmic units of each hospital. The number of patients was estimated from the OPD registry of the ophthalmic unit of DMCSH and FHCSH. According to the data, 247 DMCSH patients and 210 FHCSH glaucoma patients were referred during the same period last year (March 1st to April 30th). Then, 414 patients were consecutively included in this study. However, just 92.3% of participants responded (382). Consequently, the results were inferred from 382 participants.

### Variables

#### Dependent variable

Glaucoma treatment adherence.

#### Independent variables

Socio-demographic characteristics (age, sex, residence, educational level, marital status and occupation), disease-related variables (stage of disease and Time of onset of disease), therapy-related variables (drug side effects, type, number & frequency of medications, drugs for other diseases and

interval between medical visits), patient-related variables (knowledge, attitude and availability of social support), and health care system-related variables (patient-provider relationship, waiting time at follow up, scheduling problems, distance from the health institutions).

### Operational Definitions

#### Adherence

Participants were adherent when the MMAS – 8 score is < 2 and non-adherent when the MMAS – 8 score is ≥ 2 (22, 23).

#### Knowledge

Respondents were considered to have good knowledge if they scored ≥ the mean score of knowledge questions and inadequate knowledge if they scored < the mean score of knowledge questions (24, 25).

#### Favorable attitude

Those participants who were positively worded and scored points ≥ the mean value in the attitude questionnaire.

#### Unfavorable attitude

Those participants who were negatively worded and scored points < the mean in the attitude questionnaire (26, 27).

#### Social support

The Oslo score ranging between 3 and 8 was classified as ‘poor support’, a score between 9 and 11 as ‘intermediate support’, and a score between 12 and 14 as ‘strong support’ (28).

#### Distance

Short distance within 5km, intermediate within 6-10 km and long-distance >10km (29).

#### Visual acuity level

Normal/near normal vision was between 6/6-6/18 in the better eye, low vision 6/24 to count finger greater than one meter to the better eye and blind/near blindness was from count finger in front to no light perception to the better eye (2).

#### Elevated IOP

IOP>21mmHg (19).

#### Early disease

Early glaucomatous disc features (e.g., cup: disc ratio ≤ 0.65) or mild visual field defect not within 10° of fixation, or both (19).

#### Moderate disease

Moderate glaucomatous disc features (e.g., cup: disc ratio 0.7: 0.85) or moderate visual field defect not within 10° of fixation, or both (19).

#### Advanced disease

Advanced glaucomatous disc features (e.g., cup: disc ratio ≥ 0.9) or visual field defect within 10° of fixation, or both (19).

#### Long waiting time on their glaucoma follow-up visits

Waiting greater than 30 minutes.

### Data collection tools and procedure

Quantitative data was collected through chart review and interviews using a structured interviewer-administered questionnaire. The charts of each eligible patient were reviewed regarding the visual acuity, duration of follow up, the types and doses of the prescribed drugs, and stages of the disease. Each consecutive patient who fulfils the inclusion criteria was interviewed by using a structured questionnaire. The interview was conducted in a separate room after their physician visit. The questionnaires for the factors were adopted from a previous study conducted in Addis Ababa, Menelik hospital (30). Adherence to glaucoma treatment was measured by using MMAS–8 which is a medication-taking behavior scale. MMAS–8 is the latest version of the scale and has a good internal consistency (Cronbach’s α = 0.83) (31). The tool was translated to the native language of the participants and face validation was done resulted Cronbach’s a score of 0.83 which is similar with that of the original tool. For social support, the 3 item Oslo social support scale (OSS-3) was used (28). To measure attitude, there were ten questions that were modified from the Hogans drug attitude inventory-10 scale and scored on a five-point Likert scale ranging from 1 = strongly agree, 2 = agree, 3 = neutral, 4 = disagree and 5 = strongly disagree (26, 27). The data was collected from the study hospitals in two months by four ophthalmic nurses and supervised by two MSc ophthalmic nurses. A pretest on 5% of the total population was conducted prior to data collection to ensure data quality, and face validity was validated by delivering it to two experienced ophthalmologists.

### Statistical analysis

Data was entered into Epi-data version 4.2 software, then it was exported to STATA version 14.0 software for analysis. Descriptive statistics (frequencies, percentages, mean, and SD) were calculated for important variables. A logistic regression model was fitted to assess the effect of an independent variable on the dependent variable. First, bivariable analysis was computed to test the association between each independent variable and the dependent variables. On bivariable analysis, those variables which were found to have a P value<0.25 were entered into a multivariable logistic regression model in order to test for independent association. The strength of the association between the different independent variables in relation to the dependent was measured using odds ratios with a 95% confidence interval (CI) and P values below 0.05 were considered statistically significant. Model fitness was checked by the Hosmer-Lemeshow goodness of fit test (P = 0.57) (32). Then results were presented in text, tables, and graphs based on the types of data.

## Result

### Socio-demographic characteristics

Three hundred eighty-two patients with glaucoma were included in this study, with a response rate of 92.3%. Of the total participants, 264 (69.1%) were males. More than half of the respondents were in the age group of 41–60 years old (51.3%), with a mean age of 57.67 ± 11.87 years (**Table 1**).

**Table 1:** Socio-demographic characteristics of respondents attending at ophthalmic unit of referral hospitals in North West Ethiopia, 2019.

Variables		Response	
		Frequency (n)	Percent (%)
Sex	Male	264	69.11
	Female	118	30.89
Age group	22-40 Years	38	9.95
	41-60 Years	196	51.31
	>60 Years	148	38.74
Religion	Orthodox	346	90.58
	Muslim	30	7.85
	Protestant	6	1.57
Marital status	Single*	46	12.14
	Married	336	87.96
Place of residence	Urban	160	41.88
	Rural	222	58.12
Educational level	Can’t Read & Write	186	48.69
	Can Read And Write	108	28.27
	Primary Education	12	3.14
	Secondary Education	18	4.71
	Higher Education	58	15.18
Occupation	Farmer	222	58.12
	Government Employee	68	17.80
	Merchant	64	16.75
	Retired	16	4.19
	Other	12	3.14

### Adherence to glaucoma treatment

Out of the total 382 participants, 189 (49.5%) [95% CI: 44.4–54.5%] had adherence to their glaucoma treatment. Of those, 38.7% occasionally forgot to take their glaucoma medications, while 21.5% occasionally missed their medications for reasons other than forgetting. Forty-four participants (11.5%) discontinued medication when they felt worse, and 42 (11%) discontinued treatment when they felt their symptoms were under control. One hundred thirty-eight (36%) participants sometimes forgot to bring along their glaucoma medicine when they traveled or left home (**Table 2**).

**Table 2:** Response of study participants on the MMAS-8 attending follow up at ophthalmic unit of referral hospitals in North West Ethiopia, 2019.

MMAS-8		Frequency	Percent
Do you sometimes forget to take your glaucoma medication?	No	234	61.3
	Yes	148	38.7
People sometimes miss taking their medications for reasons other than forgetting. Thinking over the past two weeks, were there any days when you did not take your medicine?	No	300	78.5
	Yes	82	21.5
Have you ever stopped taking your medicine without telling your doctor because you felt worse when you took it?	No	338	88.5
	Yes	44	11.5
When you travel or leave home, do you sometimes forget to bring along your medicine?	No	244	64
	Yes	138	36
Did you take all your medicine yesterday?*	Yes	328	85.8
	No	54	14.2
When you feel like your symptoms are under control, do you sometimes stop taking your medicine?	No	340	89
	Yes	42	11
Taking medicine every day is a real inconvenience for some people. Do you ever feel hassled about sticking to your treatment plan?	No	318	83
	Yes	64	17
How often do you have difficulty remembering to take all your medicine?	Never	156	40.8
	Once in a while	6	1.6
	Sometimes	196	51.3
	Usually	16	4.2
	All the time	8	2.1

### Patient related factors

**Figure 1:**
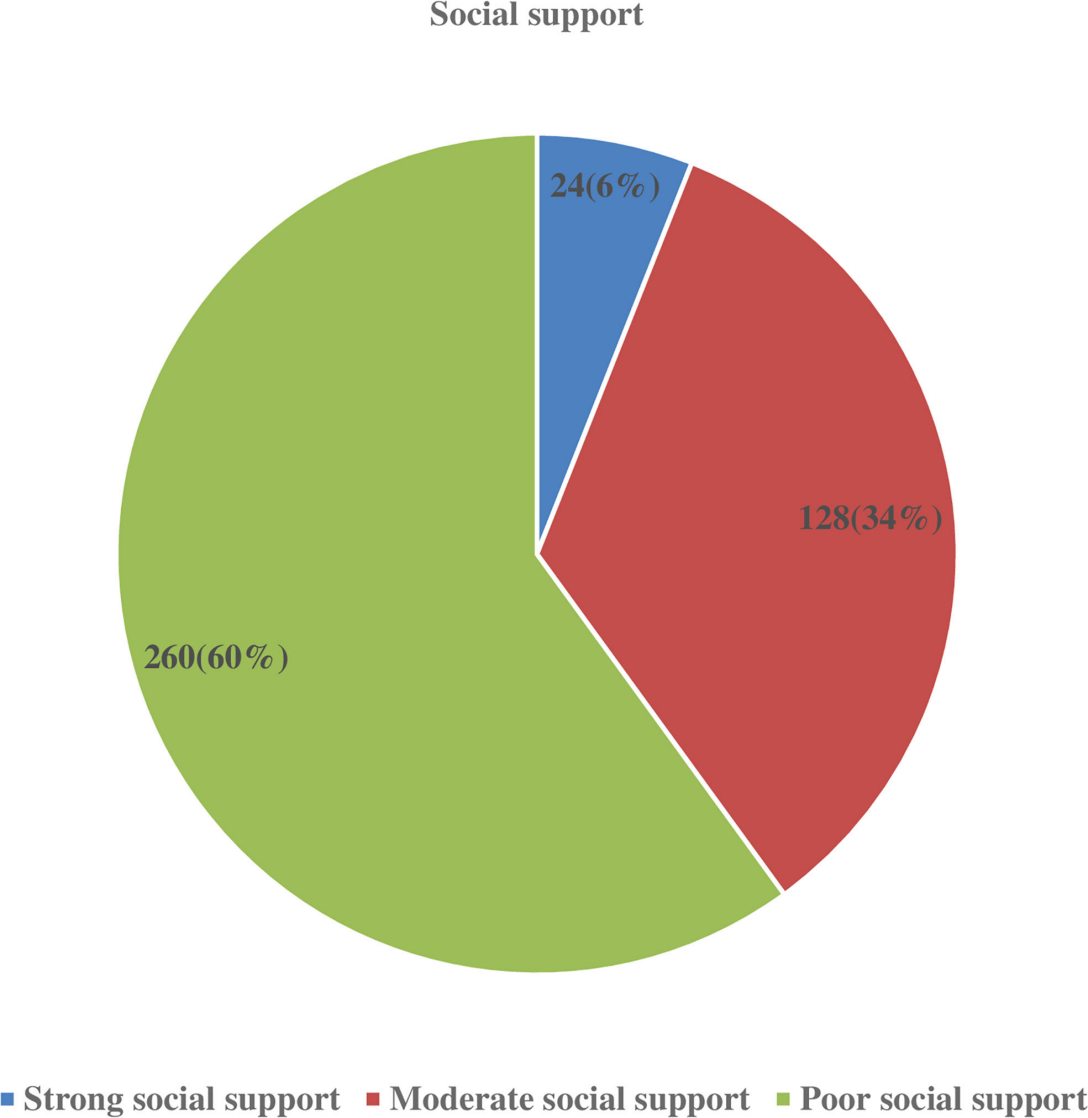
Level of social support among glaucoma patients attending ophthalmic follow up units of referral hospitals in North West Ethiopia, 2019.

Overall, 222 (58.1%) participants had good knowledge regarding glaucoma symptoms and risk factors. The mean knowledge score for glaucoma was 4.3 ( ± 2 SD). The majority of respondents (85.9%) knew that glaucoma is a blinding eye disease (**Table 3**). Out of the total participants, 294 (77%) had a favorable attitude towards their glaucoma treatments (**Table 5**) and 230 (60.2%) had poor social support (**Figure 1**). The study participants were also interviewed for certain enabling factors to take their glaucoma medications. The majority of respondents (326 [85.4%]) stated that the fear of going blind is a major motivator for them to take their glaucoma medications (**Table 4**).

**Table 3:** The response of participants on each knowledge questions and their score among glaucoma patients attending ophthalmic follow up units of referral hospitals in North West Ethiopia, 2019.

Knowledge questions		Frequency (n)	Percent (%)
Glaucoma is a blinding eye disease	Yes	328	85.9%
	No	54	14.1%
Glaucoma occurs without symptoms	Yes	242	63.4%
	No	140	34.6%
Glaucoma is often associated with high pressure in the eye	Yes	228	59.7%
	No	154	40.3%
Glaucoma runs in families	Yes	118	30.9%
	No	264	69.1%
Major vision loss from glaucoma can be prevented with treatment	Yes	246	64.4%
	No	136	35.6%
Hypertension is a risk factor	Yes	76	19.9%
	No	306	80.1%
DM is a risk factor	Yes	92	24.1%
	No	290	75.9%
Old age is a risk factor	Yes	312	81.7%
	No	70	18.3%
The score of participants out of eight knowledge questions	Score		
	0	18	4.71
	1	20	5.24
	2	52	13.61
	3	34	8.90
	4	36	9.42
	5	126	32.98
	6	44	11.52
	7	38	9.95
	8	14	3.66
	Total	382	100.00

**Table 4:** Patient related factors among glaucoma patients attending at ophthalmic unit of referral hospitals in North West Ethiopia, 2019.

	Variable		Frequency	Percent
	Living with	Spouse	158	41.4
		Children	18	4.7
		spouse & children	174	45.5
		living alone	30	7.8
		Other	2	0.05
	Attitude	Favorable attitude	294	77
		Unfavorable attitude	88	23
Enabling factors	Fear of going blind	Yes	326	85.4
		No	56	14.6
	Desire for improved vision	Yes	104	27.2
		No	278	72.8
	Knowledge of disease and importance of the drug	Yes	46	12
		No	336	88
	Convenience of drug schedule	Yes	6	1.6
		No	376	98.4
	Availability of drugs	Yes	14	3.7
		No	368	96.3
	Alarm system	Yes	12	3.1
		No	370	96.9

### Health care system related factors

A large majority of participants (368, or 96.3%) trusted their eye doctors most to give them information about their eyes. Three hundred sixty-six participants (95.8%) suffered from a long waiting time on their glaucoma follow-up visits. Of the total participants, 132 (34.5%) had problems in scheduling times for their glaucoma treatment visits. The majority of participants who traveled a short distance (<5km) for their glaucoma treatment follow-up had good adherence (75%). However, only 33% had good adherence among long-distance travelers (>10km) (**Table 5**).

**Table 5:** Health care system related factors among glaucoma patients attending at ophthalmic unit of referral hospitals in North West Ethiopia, 2019.

		Response	
		Frequency	100 %
Do you trust eye doctors most to give them information about their eyes?	Yes	368	96.3
	No	14	3.7
Do you have good relationship with the ophthalmologist?	Yes	366	95.8
	No	16	4.2
Does the Doctor spends enough time with you?	Yes	350	91.6
	No	32	8.4
If you had questions about eye medicine, would ask eye doctor?	Yes	356	93.2
	No	26	6.8
Do Ophthalmologists answer the patients’ question?	Yes	352	92.1
	No	30	7.9
If you had questions about eye medicine, would ask pharmacist?	Yes	328	85.9
	No	54	14.1
Ophthalmologist discussed the importance of taking medications as prescribed?	Yes	346	90.6
	No	36	9.4
Ophthalmologist/pharmacist explained how to use medications?	Yes	358	93.7
	No	24	6.3
Do you have problems in scheduling times for medical visits?	Yes	132	34.5
	No	250	65.4
Do you suffer from a long waiting time on your follow up?	Yes	366	95.8
	No	16	4.2
Distance from the Hospital to your home	≤ 5Kms	110	28.8
	6-10Kms	60	15.7
	>10Kms	212	55.5

### Therapy and disease related factors

Regarding the number of drugs, those participants who took only one glaucoma medication were 66 (17%). Only 30 (7.9%) of the study participants reported side effects from their glaucoma medications, and only 12 (3.1%) of those participants were taking medications for other diseases. The majority, 96.8% (n = 370), of the participants had no comorbidities. Among comorbidities, DM was the most common comorbidity that accounted for more than 50%. Most participants (328, 85.9%) had an appointment interval of every two to three months. The mean intraocular pressure (IOP) of respondents in mmHg in the right eye and left eye was 23.04 (SD: ± 11.15) and 23.72 (SD: ± 9.73) respectively. Regarding the severity of glaucoma, 16.6% (n = 63), 47.6% (n = 181) and 25.3% (n = 96) of patients had early stage, moderate and advanced glaucoma, respectively. Patients had been diagnosed with glaucoma for an average of 3.4 years (SD ± 3.1 years; range: 6 months to 25 years). Almost all participants were taking timolol 2% two times per day, and most participants were taking pilocarpine 0.5% four times per day for their glaucoma treatments. Two hundred forty-eight (64.9%) participants had community-based health insurance. Regarding the level of visual acuity, less than one third of the patients (n = 110, 28.8%) had normal or near normal vision (**Table 6**).

**Table 6:** Therapy and disease related factors among glaucoma patients attending at ophthalmic unit of referral hospitals in North West Ethiopia, 2019.

		Response	
		Frequency (n)	100 (%)
How many types of anti-glaucoma medications do you take?	One	66	17.0
	Two	276	71.0
	Three	40	10.3
Do you have side effects from glaucoma medications?*	Yes	30	7.9
	No	352	92.1
Do you take medications for other diseases?	Yes	12	3.1
	No	370	96.9
Do you have community based health insurance (CBHI)?	Yes	248	64.9
	No	134	35.1
Cost of glaucoma medications	Affordable	26	19.7
	Not affordable	106	80.3
Disease onset(in months)	6-24 months	180	47.1
	25-60 months	142	37.2
	>=61 months	60	15.7
What is the interval for your glaucoma treatment follow up visits?	<= every month	48	12.6
	Every 2-3 months	328	85.9
	> 3 months	6	1.6
Severity of glaucoma disease	Elevated IOP	40	10.5
	Early disease	63	16.6
	Moderate disease	181	47.6
	Advanced disease	96	25.3
Level of Visual Acuity	Normal/near normal VA (6/6 - 6/18)	190	49.7
	Low vision (6/24 - 6/60 to CF>1m)	166	43.5
	Near blindness/blind (CF in front to NLP	26	6.8

### Factors associated with glaucoma treatment adherence

In this study, occupation, knowledge level, attitude, distance from the hospitals, and scheduling problems for glaucoma medical follow-up visits were found to be potential factors of adherence to glaucoma treatments. This study showed that patients with the occupation of merchant were 80% less likely to adhere to their glaucoma treatment as compared to farmers [95% CI (0.08, 0.52), P < 0.001]. Patients with good knowledge were 2.24 times more likely to adhere to their glaucoma treatment as compared to patients with inadequate knowledge [95% CI (1.20, 4.20), P=0.012]. This study also revealed that participants with a favorable attitude were 5.77 times more likely to adhere to their glaucoma treatment as compared to participants with an unfavorable attitude [95% CI (2.22, 15.00), P < 0.001]. Patients who traveled a short distance (5km) and a medium distance (6–10km) to the hospitals were twice and three times more likely to adhere to their glaucoma treatments, respectively, than patients who traveled a long distance (> 10km) [95% CI (4.25, 28.22), P < 0.001] and [95% CI (1.41, 6.67), < 0.001] (**Table 7**).

**Table 7:** Bivariable &Multivariable logistic regression analysis of factors associated with treatment adherence among patients attending the ophthalmic unit of referral hospitals in North West Ethiopia, 2019.

Variables	Adherence		COR	AOR (95% CI)	P Value
	Adherence, n (%)	Non-adherence, n (%)			
Occupation					
Farmer	97 (43.7)	125 (56.3)	1.00	1.00	
Gov’t employee	46 (67.7)	22 (32.3)	2.69	0.48 (0.17, 1.32)	0.157
Merchant	26 (40.6)	38 (59.4)	0.88	0.20 (0.08, 0.52)	0.001*
Other	20 (71.4)	8 (28.6)	3.22	0.83 (0.21, 3.34)	0.804
Fear of going blind					
Yes	176 (54)	150 (46)	3.88	1.35 (0.56,3.24)	0.499
No	13 (23.2)	43 (76.8)	1.00	1.00	
Knowledge about glaucoma (symptoms and risk factors)					
Good knowledge	153 (68.9)	69 (31.1)	7.63	2.24 (1.20, 4.20)	0.012*
Inadequate knowledge	36 (22.5)	124 (77.5%)	1.00	1.00	
Attitude					
Favorable attitude	182 (62)	112 (38%)	18.8	5.77 (2.22, 15.00)	0.001*
Unfavorable attitude	7 (7.9)	81 (92.1)	1.00	1.00	
Distance from the hospital					
<=5km	82 (74.5)	28 (25.5)	5.94	10.96 (4.25,28.22)	0.001*
6-10km	37 (61.7)	23 (32.3)	3.26	3.07 (1.41, 6.67)	0.005*
>10Km	70 (33)	142 (67)	1.00	1.00	
Scheduling time problems for medical follow up visits					
Yes	24 (18.2)	108 (82.8)	0.11	0.18 (0.09, 0.36)	0.001*
No	165 (66)	85 (34)	1.00	1.00	

## Discussion

Adherence refers to how well people follow their doctors’ orders when taking drugs. For the sake of their health, glaucoma patients should take their medications based on physicians the order. According to previous research conducted around the world, adherence to glaucoma therapy is not satisfactory (3). The present study showed that the adherence level was 49.5% [95% CI (44.4, 54.5%)]. This is consistent with studies done by the World Health Organization that reported adherence rates of 50% to long-term therapies among patients suffering from chronic diseases in the general population (33), north India 51.66% [16] and in Egypt 46.4% (12). However, the adherence rate of the current study was higher than the adherence rate of other studies done in USA 40% (8), Iran 32.5% (13), Menelik II referral hospital(42.6%) (2) and Jima University Specialized Hospital (32.5%) (16). This variation might be partly attributable to the methodological difference (exploratory study (USA) and tool difference (MMAS-4 in Iran). On the other hand, the adherence level of this study was found to be lower than other studies done in Canada (72.1%) (9) and Dutch (72.7%) (10). This difference might be attributable to inconsistency in the definition of adherence/non-adherence and geographical and other socio-economic differences.

Patients with merchant occupations were 80% less likely to adhere to their glaucoma treatment than patients with farmer occupations [95% CI (0.08, 0.52)]. In contrast to this finding, a study done at Menelik II Referral Hospital revealed that farmer occupation was significantly associated with non-adherence (2). Perhaps this variation might be partly due to the use of community-based health insurance and due to people forgetting to bring along their glaucoma medicine when they travel or leave home. Merchants usually travel from place to place, and this behavior might lead to forgetting their glaucoma medications.

This study found that patients with good knowledge were 2.24 times more likely to adhere to their glaucoma treatment as compared to patients with inadequate knowledge [95%CI (1.20, 4.20)]. This finding was supported by studies done in Egypt (12) and Nigeria (25). Patients with glaucoma who stick to their therapy more than 80% rate of treatment adherence are less likely to become blind (34). This study also revealed that participants with a favorable attitude were 5.77 times more likely to adhere to their glaucoma treatment as compared to participants with an unfavorable attitude [95% CI (2.22, 15.00)]. This finding was consistent with a study done in Korea (35).

This study also found that patients who traveled a short distance (5km) and a medium distance (6–10km) to hospitals were approximately eleven and three times more likely to adhere to their glaucoma treatments, respectively, than patients who traveled a long distance (> 10km) [95 CI (4.25, 28.22) and (95% CI (1.41, 6.67)]. This is in line with studies conducted on other chronic diseases (36, 37). However, a study in Pennsylvania contrasts this finding (38).This might be due to the socio-economic differences of study participants.

Those participants who had a scheduling problem for their glaucoma follow up visits were 82% less likely to adhere compared to participants who had no scheduling problems during their glaucoma treatment follow-up visits [95%CI (0.09, 0.36)]. This finding was supported by studies conducted in Ann Arbor, Michigan (39) and Iran (13). It is obvious that if patients got appropriate time for their follow-up visit, they might easily adhere to their glaucoma treatment. This might imply that patients require informed decision-making for their treatments and follow-up visits. As treatment success depends on the optimum adherence, determination of the patient and agreement on treatment is the most important step (40).

## Limitations

Cross-sectional study design makes it difficult to draw causal relationships between dependent and independent variables/factors. Research methodologies also involved self-reported measures that largely depend on individuals’ memory, and recall bias may exist. This finding was also based on the indirect method of adherence measurement (patients’ self-report) that had its own drawbacks, particularly social desirability bias.

## Conclusion

The findings from the present study indicated that the rate of adherence to glaucoma treatments in DMRH and FHCSH was low when it is compared from previously studied researches. Some studies consider rates of greater than 80% to be acceptable, whereas others consider rates of greater than 95% to be mandatory for adequate adherence, particularly among patients with serious conditions (17). Socio-demographic factors, healthcare system-related factors such as scheduling issues for glaucoma medical follow-up visits and distance from hospitals, and patient-related factors (knowledge and attitude) were all significantly associated with glaucoma treatment adherence. It is better to enhance the informed decision-making power of patients to avoid scheduling problems for glaucoma medical follow-up visits. Assure the establishment of care delivery systems that allow nearby glaucoma treatment options, as well as a means of accurate assessment of adherence to glaucoma treatment and design strategies to improve patients’ knowledge and attitude towards glaucoma treatment adherence.

## Data availability statement

The original contributions presented in the study are included in the article/supplementary material. Further inquiries can be directed to the corresponding author.

## Ethics statement

The studies involving human participants were reviewed and approved by College of Health Sciences at Debre Markos University with a reference number of DMUCHS/0423/12/2012. The patients/participants provided their written informed consent to participate in this study.

## Author contributions

All authors listed have made a substantial, direct, and intellectual contribution to the work and approved it for publication.

## Acknowledgments

The authors would like to thank the study participants for their cooperation and the Debre Markos and Felege Hiwot hospital staff for their support.

## Conflict of interest

The authors declare that the research was conducted in the absence of any commercial or financial relationships that could be construed as a potential conflict of interest.

## Publisher’s note

All claims expressed in this article are solely those of the authors and do not necessarily represent those of their affiliated organizations, or those of the publisher, the editors and the reviewers. Any product that may be evaluated in this article, or claim that may be made by its manufacturer, is not guaranteed or endorsed by the publisher.

## Abbreviations

AOR, Adjusted odds Ratio; CBHI, Community Based Health Insurance; CI, Confidence Interval; COAG, Chronic Open Angle Glaucoma; COR, Crude Odds Ratio; DMCSH, Debre Markos Comprehensive Specialized Hospital; DMU, Debre Markos University; FHCSH, Felege Hiwot Comprehensive Specialized Hospital; IOP, Intraocular pressure; MMAS-8, Morisky Medication Adherence Scale-8; OD, Oculus Dexter; OS, Oculus Sinister; OU, Oculus Unitas; WHO, World Health Organization; VA, Visual Acuity.

## References

[1] Brunner LS . Brunner & suddarth's textbook of medical-surgical nursing. Philadelphia: Lippincott Williams & Wilkins (2010).

[2] Mehari T Giorgis AT Shibeshi W . Level of adherence to ocular hypotensive agents and its determinant factors among glaucoma patients in menelik II referral hospital, Ethiopia. BMC Ophthalmol (2016) 16(1):131. doi: 10.1186/s12886-016-0316-z 27485739 PMC4969714

[3] Tham Y-C Li X Wong TY Quigley HA Aung T Cheng C-Y . Global prevalence of glaucoma and projections of glaucoma burden through 2040: a systematic review and meta-analysis. Ophthalmology (2014) 121(11):2081–90. doi: 10.1016/j.ophtha.2014.05.013 24974815

[4] Pascolini D SPM . Global estimates of visual impairment. Br J Ophthalmol (2012) 96(5):614–8. doi: 10.1136/bjophthalmol-2011-300539 22133988

[5] Kyari F Abdull MM Bastawrous A Gilbert a CE Faal H . Epidemiology of glaucoma in sub-Saharan Africa: prevalence, incidence and risk factors. Middle East Afr J Ophthalmol (2013) 20:111–25. doi: 10.4103/0974-9233.110605 PMC3669488 23741130

[6] Atey TM Shibeshi W T Giorgis A Asgedom SW . The impact of adherence and instillation proficiency of topical glaucoma medications on intraocular pressure. J Ophthalmol (2017) 2017. doi: 10.1155/2017/1683430 PMC5618783 29104803

[7] Ebeigbe J . Glaucoma medication adherence in an adult population in Nigeria. Afr J Med Health Sci (2017) 16:12–8. doi: 10.4103/ajmhs.ajmhs_80_16

[8] Stryker JOE Beck AD Primo SA Echt KV Bundy L Pretorius GC . An exploratory study of factors influencing glaucoma treatment adherence. J Glaucoma (2010) 19(1):66–72. doi: 10.1097/IJG.0b013e31819c4679 20075676 PMC2808197

[9] Kholdebarin R Campbell RJ Jin Y-P Buys YM Group CCS . Multicenter study of compliance and drop administration in glaucoma. Can J Ophthalmol (2008) 43(4):454–61. doi: 10.3129/i08-076 18711461

[10] Olthoff CM Hoevenaars JG van den Borne BW Webers CA Schouten JS . Prevalence and determinants of non-adherence to topical hypotensive treatment in Dutch glaucoma patients. Graefe's Arch Clin Exp Ophthalmol (2009) 247(2):235. doi: 10.1007/s00417-008-0944-y 18802720

[11] Rajurkar K Dubey S Gupta PP Denny J Chauhan L . Compliance to topical anti-glaucoma medications among patients at a tertiary hospital in north India. J Curr Ophthalmol (2017) 30:125–9. doi: 10.1016/j.joco.2017.09.002 PMC6033778 29988888

[12] Hussein NBA Eissa I Abdel-Kader AA . Analysis of factors affecting patients’ compliance to topical antiglaucoma medications in Egypt as a developing country model. Hindawi Publishing Corporation (2015) 2015:7. doi: 10.1155/2015/234157 PMC4488247 26167292

[13] Movahedinejad T Adib-Hajbaghery M . Adherence to treatment in patients with open-angle glaucoma and its related factors. Electronic Physician (2016) 8(9):2954. doi: 10.19082/2954 27790350 PMC5074756

[14] Ngozi oNwubiko S zuada Nwachukwu N . Glaucoma medications: issues with adherence in a tertiary hospital in Nigeria. Family Med Primary Care Rev (2020) 22(4):302–6. doi: 10.5114/fmpcr.2020.100436

[15] Singh L Sharma A Chaturvedi A . Socio demographic profile of glaucoma patients and barriers to treatment compliance. Indian J Clin Exp Ophthalmol (2020) 6(1):17–21. doi: 10.18231/j.ijceo.2020.006

[16] Tamrat L Gessesse GW Gelaw Y . Adherence to topical glaucoma medications in Ethiopian patients. Middle East Afr J Ophthalmol (2015) 22(1):59. doi: 10.4103/2F0974-9233.148350 25624675 PMC4302478

[17] Osterberg L Blaschke T . Adherence to medication. New Engl J Med (2005) 353(5):487–97. doi: 10.1056/NEJMra050100 16079372

[18] Lam WY Fresco P . Medication adherence measures: An overview. BioMed Res Int (2015) 2015:217047. doi: 10.1155/2015/217047 26539470 PMC4619779

[19] Damji KF Behki R Wang L . Canadian Perspectives in glaucoma management: setting target intraocular pressure range. Can J Ophthalmol J Canadien d'ophtalmologie. (2003) 38(3):189–97. doi: 10.1016/S0008-4182(03)80060-1 12733686

[20] Dreer LE Girkin C Mansberger SL . Determinants of medication adherence to topical glaucoma therapy. J Glaucoma (2012) 21(4):234. doi: 10.1097/IJG.0b013e31821dac86 21623223 PMC3183317

[21] Castel OC Keinan-Bokerb L Geyer O Milmana U Karkabia K . Factors associated with adherence to glaucoma pharmacotherapy in the primary care setting. Family Practi (2014) 31(4):453–61. doi: 10.1093/fampra/cmu031 PMC4106405 24927725

[22] Muntner P Joyce C Holt E He J Morisky D Webber LS . Defining the minimal detectable change in scores on the eight-item morisky medication adherence scale. Ann Pharmacother (2011) 45(5):569–75. doi: 10.1345/aph.1P677 21521862

[23] Ribeiro MVMR Ribeiro LEF Ribeiro ÊAN Ferreira CV Barbosa FT . Adherence assessment of eye drops in patients with glaucoma using 8 item morisky score: a cross sectional study. Bras Oftalmol (2016) 45:569–75. doi: 10.5935/0034-7280.20160087

[24] Tadesse F Mulugeta A . Compliance to topical anti-glaucoma medication among glaucoma patients at menelik II tertiary hospital, Addis Ababa. Ethiopia (2014) 6:432–7.

[25] Ogbonnaya CE Ogbonnaya LU Okoye OA Kizor-Akaraiwe N . Glaucoma awareness and knowledge, and attitude to screening, in a rural community in ebonyi state, Nigeria. Open J Ophthalmol (2016) 6:119–27. doi: 10.4236/ojoph.2016.62017

[26] Hogan TP Awad A Eastwood R . A self-report scale predictive of drug compliance in schizophrenics: reliability and discriminative validity. psychol Med (1983) 13(1):177–83. doi: 10.1017/S0033291700050182 6133297

[27] Stjernsward S Persson K Nielsen R Tuninger E Levander S . A modified drug attitude inventory used in long-term patients in sheltered housing. Eur Neuropsychopharmacol J Eur Coll Neuropsychopharmacol (2013) 23(10):1296–9. doi: 10.1016/j.euroneuro.2012.11.011 23265955

[28] D OS . Explanation of OSS-3. European public health information system. ROBERT KOCH INSTITUT: EUPHIX (2008).

[29] Tegegne TK Chojenta C Loxton D Smith R Kibret KT . The impact of geographic access on institutional delivery care use in low and middle-income countries: Systematic review and meta-analysis. PloS One (2018) 13(8):e0203130. doi: 10.1371/journal.pone.0203130 30161201 PMC6117044

[30] Tadesse F Mulugeta A . Compliance to topical anti-glaucoma medication among glaucoma patients at menelik II tertiary hospital, Addis Ababa, Ethiopia. Ethiopian J Health Dev (2015) 29(1):1–79.

[31] Morisky DE Ang A Krousel-Wood M Ward HJ . Predictive validity of a medication adherence measure in an outpatient setting. J Clin Hypertension (Greenwich Conn) (2008) 10(5):348–54. doi: 10.1111/j.1751-7176.2008.07572.x PMC2562622 18453793

[32] Hosmer DW Lemeshow S Klar J . Goodness-of-fit testing for the logistic regression model when the estimated probabilities are small. Biometrical J (1988) 30(8):911–24. doi: 10.1002/bimj.4710300805

[33] Sabaté E . Adherence to long−term therapies: Evidence for action. Switzerland: Switzerland WHO (2003). 14562485

[34] Kim CY Park KH Ahn J Ahn M-D Cha SC Kim HS . Treatment patterns and medication adherence of patients with glaucoma in south Korea. Br J Ophthalmol (2017) 101(6):801–7. doi: 10.1136/bjophthalmol-2016-308505 PMC5583683 28270490

[35] Hong S Kang SY Yoon JU Kang U Seong GJ Kim CY . Drug attitude and adherence to anti-glaucoma medication. Yonsei Med J (2010) 51(2):261–9. doi: 10.3349/ymj.2010.51.2.261 PMC2827921 20191020

[36] Shargie EB Lindtjørn B . Determinants of treatment adherence among smear-positive pulmonary tuberculosis patients in southern Ethiopia. PloS Med (2007) 4(2):e37. doi: 10.1371/journal.pmed.0040037 17298164 PMC1796905

[37] Amram O Shoveller J Hogg R Wang L Sereda P Barrios R . Distance to HIV care and treatment adherence: Adjusting for socio-demographic and geographical heterogeneity. Spatial Spatio Temporal Epidemiol (2018) 27:29–35. doi: 10.1016/j.sste.2018.08.001 30409374

[38] Pednekar P Peterson A Heller D Brown T . Does the distance to the nearest pharmacy affect medication adherence rates among elderly patients with diabetes? Value Health (2017) 20(9):A483. doi: 10.1016/j.jval.2017.08.477

[39] Newman-Casey PA Robin AL Blachley T Farris K Heisler M Resnicow K . The most common barriers to glaucoma medication adherence: A cross-sectional survey. Ophthalmology (2015) 122(7):1308–16. doi: 10.1016/j.ophtha.2015.03.026 PMC4485580 25912144

[40] Worku K . National comprehensive HIV prevention, care and treatment training for health care providers, participant manual. Ministry Health Ethiopia (2017) 27:29–35. doi: 10.1186/s12978-017-0323-4

